# Renal sinus fat and renal hemodynamics: a cross-sectional analysis

**DOI:** 10.1007/s10334-019-00773-z

**Published:** 2019-08-31

**Authors:** Karlinde A. Spit, Marcel H. A. Muskiet, Lennart Tonneijck, Mark M. Smits, Mark H. H. Kramer, Jaap A. Joles, Anneloes de Boer, Daniel H. van Raalte

**Affiliations:** 1 Department of Internal Medicine, Diabetes Center, Amsterdam University Medical Centers, Location VUMC, De Boelelaan 1117, 1081 HV Amsterdam, The Netherlands; 2 Department of Nephrology and Hypertension, University Medical Center, Utrecht, The Netherlands; 3 Department of Radiology, University Medical Center, Utrecht, The Netherlands

**Keywords:** Renal sinus fat, Hypertension, Renal hemodynamics, Type 2 diabetes, Diabetic kidney disease

## Abstract

**Objectives:**

Increased renal sinus fat (RSF) is associated with hypertension and chronic kidney disease, but underlying mechanisms are incompletely understood. We evaluated relations between RSF and gold-standard measures of renal hemodynamics in type 2 diabetes (T2D) patients.

**Methods:**

Fifty-one T2D patients [age 63 ± 7 years; BMI 31 (28–34) kg/m^2^; GFR 83 ± 16 mL/min/1.73 m^2^] underwent MRI-scanning to quantify RSF volume, and subcutaneous and visceral adipose tissue compartments (SAT and VAT, respectively). GFR and effective renal plasma flow (ERPF) were determined by inulin and PAH clearances, respectively. Effective renal vascular resistance (ERVR) was calculated.

**Results:**

RSF correlated negatively with GFR (*r* = − 0.38; *p* = 0.006) and ERPF (*r* = − 0.38; *p* = 0.006) and positively with mean arterial pressure (MAP) (*r* = 0.29; *p* = 0.039) and ERVR (*r* = 0.45, *p* = 0.001), which persisted after adjustment for VAT, MAP, sex, and BMI. After correction for age, ERVR remained significantly related to RSF.

**Conclusions:**

In T2D patients, higher RSF volume was negatively associated to GFR. In addition, RSF volume was positively associated with increased renal vascular resistance, which may mediate hypertension and CKD development. Further research is needed to investigate how RSF may alter the (afferent) vascular resistance of the renal vasculature.

**Electronic supplementary material:**

The online version of this article (10.1007/s10334-019-00773-z) contains supplementary material, which is available to authorized users.

## Introduction

Obesity is recognized as a heterogeneous condition, in which individuals with similar levels of body mass index (BMI) may have distinct metabolic, cardiovascular (CV), and renal risk [1]. Variation in body fat distribution provides a potential explanation for some of these observations. As such, excess visceral adipose tissue (VAT) compared to subcutaneous adipose tissue (SAT) is particularly associated with the presence of adverse metabolic risk factors, CV disease, and chronic kidney disease (CKD) [2]. In addition to VAT, ectopic fat accumulation, i.e., storage of fat in non-adipose tissues, is also related to organ dysfunction [3]. A well-known example is intrahepatic lipid accumulation leading to hepatic dysfunction [4]. More recently, accumulation of fat in the renal sinus, a compartment located at the medial border of the kidney that contains renal vessels, calices, nerve fibers, and lymphatic channels, was suggested to increase risk of renal disease [5, 6]. As such, in cross-sectional studies involving overweight individuals with and without type 2 diabetes (T2D), accumulation of renal sinus fat (RSF) was shown to independently associate with reduced estimated glomerular filtration rate (eGFR) [7, 8] and CKD (defined as eGFR < 60 mL/min/1.73 m^2^ [9, 10].

Several mechanisms have been put forward to explain this association. First, RSF has been associated with systemic hypertension, which is a driver of CKD [11, 12]. Notably, this association is independent of other adipose tissue compartments [7, 11, 13]. Second, by compressing various renal structures, reduced tissue perfusion and tubular flow may occur [8]. Third, in analogy to adipocytes from perivascular adipose tissue (PVAT), RSF adipocytes may secrete pro-inflammatory adipokines leading to renal inflammation, fibrosis and dysfunction [7, 14, 15]. Fourth, RSF could modulate renal hemodynamics by secreting vasoconstrictive factors, as several studies have demonstrated PVAT to modulate vascular tone and skeletal muscle perfusion [16]. Whether this mechanism of altered renal hemodynamics is present in T2D patients remains unknown. Therefore, we aimed to evaluate the role of RSF on renal hemodynamic regulation in overweight patients with T2D with normal kidney function.

## Research design and methods

This was a cross-sectional post hoc analysis of the SAFEGUARD study, which was originally designed to assess the safety of incretin-based therapies [17]. In the current analysis, T2D patients were included if magnetic resonance imaging (MRI) of the abdomen and measurements of renal hemodynamics were performed at baseline. The study was approved by the medical ethics committee of the VU University Medical Center (Amsterdam, The Netherlands), registered at ClinicalTrials.gov (NCT01744236) and was conducted in accordance with the Declaration of Helsinki and the International Conference on Harmonization of Good Clinical Practice. Written informed consent was obtained from all participants prior to performing any study-specific activity [17, 18].

### Study population

Inclusion and exclusion criteria were reported previously [17]. In brief, patients were Caucasian men or postmenopausal women, aged 35–75 years, with T2D (HbA1c 6.5–9.0%; treated with a stable dose of metformin and/or sulfonylurea) and a BMI 25–40 kg/m^2^. Key exclusion criteria included a history of malignancy, active or recent (<6 months) cardiovascular disease, eGFR < 60 mL/min/1.73 m^2^, current nephritis, urinary tract infection or urinary retention (as assessed by bladder ultrasonography at the screening-visit), allergy to any of the test substances, or inability to undergo MRI.

### Study protocol

Each patient underwent one MRI scan and 1 renal testing day as indicated below, planned on 2 separate days in random order and performed with median (interquartile range) 5 (4–9) days apart. Measurements were performed after an overnight fast, and all patients delayed their morning medication, except for metformin and thyroid hormone replacement therapy. Prior to the renal tests, blood pressure was measured three times with an oscillometric blood pressure measurement device (Dinamap^®^, GE Healthcare, Little Chalfont, United Kingdom) and the last two measurements were averaged. The study protocols were published previously [17–19] and are summarized below.

#### Renal measurements

GFR and effective renal plasma flow (ERPF) were determined by gold-standard inulin and para-amino hippuric acid (PAH) renal clearance methodology, respectively, based on timed urine sampling as published. The effective renal blood flow (ERBF) was calculated from the ERPF by dividing this by (1-hematocrit). From these values, filtration fraction (FF) and effective renal vascular resistance (ERVR) were calculated as follows: FF = GFR/RPF and ERVR = MAP/ERBF, with MAP being mean arterial pressure [19]. Renal damage markers consisted of albumin/creatinine ratio (ACR), kidney injury molecule-1/creatinine ratio (KIM-1), and neutrophil gelatinase-associated lipocalin/creatinine ratio (NGAL), all measured in urine. The fractional excretion of sodium (FE_NA_) was calculated with inulin as reference substrate.

#### Imaging protocol

The MRI protocol was focused on the pancreas, but included anatomical T_1_-weighted dual-phase gradient-echo images of the kidney (scan parameters in Table 1). MRI scans were performed by trained research physicians (MS and LT) with patients in a supine position using a 1.5-T MRI system, with a phased-array body-coil (Magnetom Avanto, Siemens Healthcare, Erlangen, Germany). The MRI protocol included an axial T1-weighted dual-phase gradient-echo sequence (“in-/opposed-phase”) with 6 mm slice thickness and a 7.8 mm gap (other MRI features are displayed in Table 1).

**Table 1:** MRI scan parameters

	In/opp phase
Sequence	T1-weighted gradient echo
TE (ms)	2.38/4.76
TR (ms)	100
Flip angle (°)	70
Pixel bandwidth	476
Acquisition time (s)	2 × 15 (breath holding)
Number of slices	24
Slice thickness (mm)	6
Slice gap (mm)	7.8
Field of view (mm)	262 × 350
Matrix size	154 × 256
Orientation	Axial
Half Fourier	0.8

*TE* echo time, *TR* repetition time

### Renal sinus fat MRI analysis

Since the imaging protocol was focused on the pancreas, sometimes, the caudal part of the kidneys was missing. Therefore, a single-slice analysis was performed as proposed by Foster et al. [20]. To assure and standardize RSF measurement at the same level for each patient, we selected the slice on which the renal artery entered the kidney. As two studies found an asymmetrical distribution of RSF (left kidney accumulates significantly more adipose tissue than the right kidney), Krievina et al*.* recommend to assess the RSF in the left kidney rather than in the right one, stating that changes in RSF were more reliably observed this way [21, 22]. For these reasons, we selected only the left kidney for further analyses.

We validated our single-slice protocol by selecting a subgroup of 15 patients whose scans displayed the complete left kidney, after which we calculated the 3D RSF volume by multiplying the surface of the RSF area (cm^2^) by the slice thickness to obtain the volume in cm^3^ on every slice. We then adjusted for the interslice gaps using the mean of the adjacent slice images (corrected for interslice thickness) and finally summed the individual volumes of the slices to acquire the 3D RSF volume. Correlations and Bland–Altman plot of the single-slice RSF area with the 3D RSF volume in these 15 patients demonstrated that the single-slice RSF is an accurate representation for the full 3D RSF volume (for the validation data of this subgroup of 15, see Supplementary files). The same approach was used to validate single-slice representation of the total kidney (TK) area in these 15 patients. Furthermore, to exclude a systematic bias in RSF measurements due to different kidney sizes, we calculated the ratio of the RSF to total kidney size (RSF/TK), as done by others [10, 22].

#### Image analysis

All MRI scans were managed and interpreted on a dedicated terminal by a single experienced investigator (KS), who was blinded to the results of the renal tests. The Slice-O-Matic 5.0 Rev-6c (TomoVision) software analysis program was used for all the fat compartment measurements. The scans were first evaluated for eligibility for this study; patients were excluded when they presented with cysts or other structural abnormalities in the renal sinus of the left kidney or when their MRI scan was of insufficient quality to properly assess RSF.

**Fig. 1:**
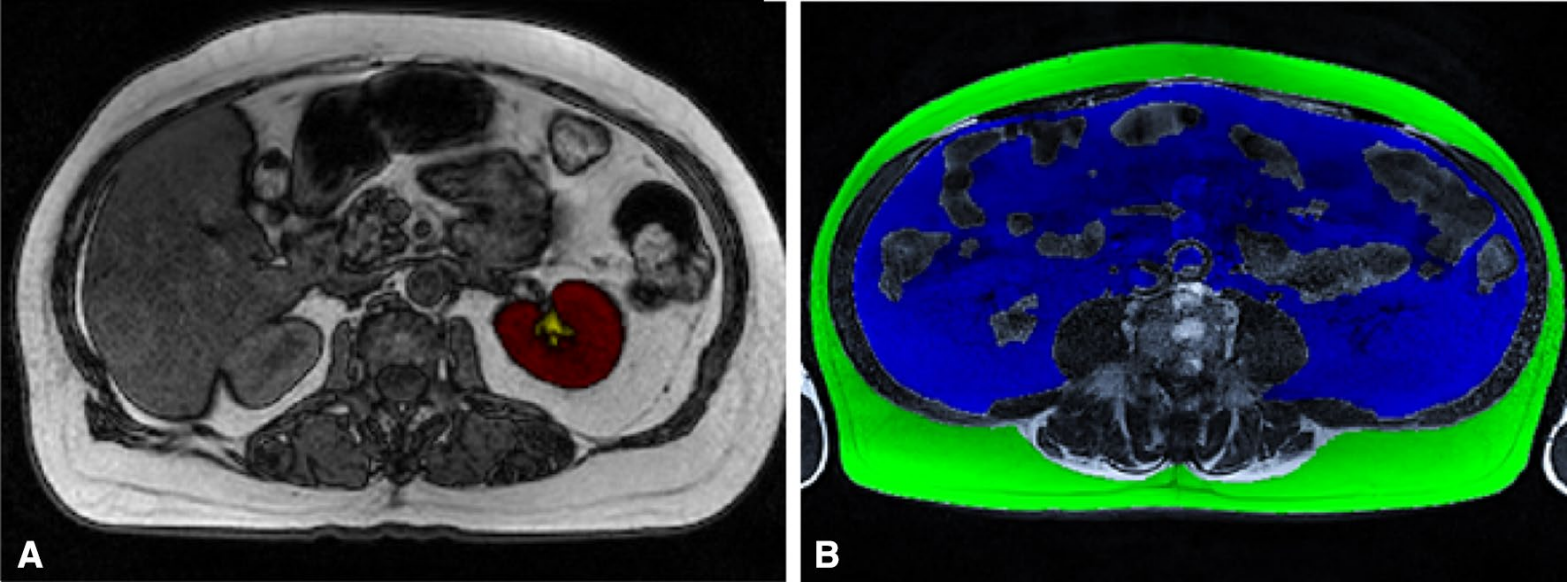
**a** Single MRI slice of RSF measurement. The total area of the left kidney (TK, red) and RSF (yellow) were manually traced and calculated. The renal artery and vein were traced to ensure they were excluded from the RSF measurements. **b** MRI scan of SAT (green) and VAT (blue) measurement

For the RSF measurements, the opposed-phase images were used. The area of the renal parenchyma was manually traced and then calculated (TK). Then, the area of the renal artery and vein in the sinus was traced in a different color to ensure that they were excluded from the RSF measurement. Next, the RSF area was colored and calculated within the curvature of the kidney (Fig. 1a). On the three abdominal T1-weighted abdominal slices, the subcutaneous adipose tissue (SAT) and VAT compartments were assessed. The outer borders of the SAT and VAT areas were drawn, after which the full SAT/VAT surface could be colored and calculated (Fig. 1b). The values of the three slices were summed and averaged for both SAT and VAT.

Intra-observer variability and inter-day variation for both RSF and SAT/VAT measurements were determined by calculating the intra-class correlation coefficient (ICC) of 24 scans in 12 patients [18]. The ICCs of the intra-observer variability and inter-day variation of RSF were 0.93 and 0.90, respectively. The ICCs of the intra-observer variability and inter-day variation of the other fat compartments (i.e., SAT and VAT areas) were 0.99 and 0.86, respectively.

### Laboratory measurements

Venous blood was obtained for measurement of plasma renin concentration (PRC) (for which the patients were in a supine position for a minimum of 20 min), glucose, and HbA1c (assays have been described) [18]. The renal damage markers were measured using immunonephelometric methods for ACR and ELISA analyses for KIM-1 and NGAL.

### Statistical analysis

RSF and RSF/TK were not normally distributed; log transformation was applied for both variables before analyses. Pearson or Spearman correlation coefficients, as appropriate, were used to assess univariate correlations between RSF and (intra-)renal hemodynamic parameters. Then, correlations between RSF and factors known to influence either RSF or renal hemodynamics were assessed. Potential effect modification on RSF/TK was assessed for BMI, sex, age, ACR, and mean arterial pressure (MAP). Multivariate linear regression models were used to assess the significance of covariate-adjusted cross-sectional relations between RSF/TK and (intra-)renal hemodynamics. In the regression models, log-transformed RSF/TK was added as dependent variable and (intra-)renal hemodynamic parameters as independent variables. In addition, variables demonstrating univariate associations with a *P *value < 0.1 qualified as an independent variable for inclusion into the final model in a stepwise manner. A two-sided *P* value < 0.05 was considered statistically significant for all analyses. All statistical analyses were performed using SPSS Statistics 22.0 software (IBM SPSS, Chicago, IL, USA).

## Results

Of the 54 patients who underwent both baseline testing days, 3 patients were excluded, because they presented with renal cysts (*n* = 1) or because their MRI scan was of insufficient quality to allow assessment of RSF (*n* = 2). Baseline characteristics are described in Table 2. In general, the study population consisted of predominantly obese T2D patients with reasonable glycemic control and without advanced CKD.

**Table 2:** Clinical characteristics, fat compartments, and renal parameters

Clinical characteristics	All (*n* = 51)
Age, years	62.9 ± 6.9
Male, *n* (%)	39 (76)
BMI, kg/m^2^	31.0 (28.3–33.6)
Waist circumference, cm	101 ± 10.4
Systolic blood pressure, mmHg	139 ± 15.8
Diastolic blood pressure, mmHg	79 ± 7.4
Mean arterial pressure, mmHg	111.7 ± 10.8
Antihypertensive medication use, *n* (%)	34 (67)
RAAS inhibitor use, *n* (%)	32 (63)
Diabetes duration, years	7 (4–12)
Fasting plasma glucose, mmol/L	8.3 ± 1.5
HbA_1c_, %	7.3 ± 0.7
Plasma renin concentration, pg/mL	9.6 (4.1–22.7)
Fat compartments	
RSF, cm^2^	2.3 (1.5–3.3)
Total kidney (TK) area, cm^2^	23.3 ± 4.3
RSF/TK	0.11 (0.7–0.14)
SAT area, cm^2^	275 (222–329)
VAT area, cm^2^	263 ± 93
Renal parameters	
eGFR (MDRD), mL/min/1.73 m^2^	86 (75–98)
eGFR (CKD-EPI), mL/min/1.73 m^2^	90 (80–106)
GFR (inulin), mL/min/1.73 m^2^	83 ± 16
ERPF, mL/min/1.73 m^2^	358 ± 90
FF, %	23.7 ± 2.9
FE_Na_, %	1.16 (0.95–1.32)
NGAL, ng/mmol	6.52 (3.92–15.48)
KIM-1, ng/mmol	0.41 (0.19–1.21)
ACR, mg/mmol	1.00 (0.46–2.93)

Values are means (±SD) or medians (interquartile range)

*ACR* albumin/creatinine ratio, *BMI* body mass index, *CKD-EPI* Chronic Kidney Disease Epidemiology Collaboration, *eGFR* estimated glomerular filtration rate, *ERPF* effective renal plasma flow, *FE*_*Na*_ fractional excretion of sodium, *FF* filtration fraction, *GFR* glomerular filtration rate, *HbA1c* glycated hemoglobin, *KIM-1* kidney injury molecule-1, *MDRD* modification of diet in renal disease, *NGAL* neutrophil gelatinase-associated lipocalin, *RSF* renal sinus fat, *SAT* subcutaneous adipose tissue, *VAT* visceral adipose tissue

### Renal sinus fat and its associations

**Fig. 2:**
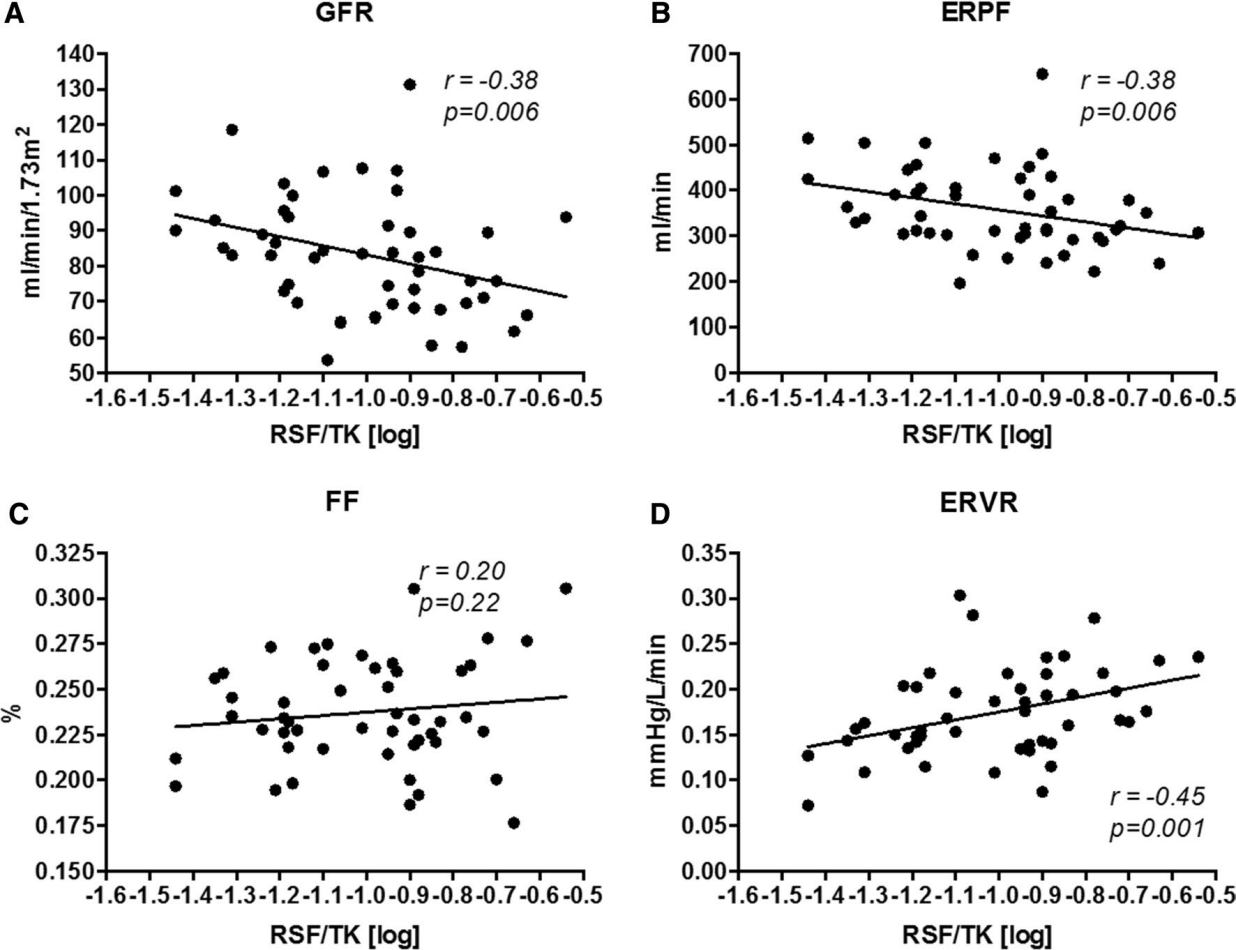
Scatterplots of RSF/TK with renal hemodynamics. *ERPF* effective renal plasma flow, *ERVR* effective renal vascular resistance, *FF* filtration fraction, *GFR* glomerular filtration rate, *RSF* renal sinus fat

RSF ranged from 0.69 to 6.95 cm^2^, with a median RSF of 2.3 cm^2^ (Table 2), and was significantly higher in male (median 2.75 cm^2^) than in female patients (1.80 cm^2^). The total kidney surface averaged 23.3 cm^2^, while the RSF corrected for TK (RSF/TK) showed a median ratio of 0.11. Scatterplots of RSF/TK and renal hemodynamics are presented in Fig. 2. Univariate correlations of RSF/TK with renal hemodynamics and renal damage markers are shown in Table 3. We performed subgroup analyses for assessment of potential effect modification and found none: consequently, all further analyses are reported for the whole group. RSF/TK demonstrated significant negative correlations with GFR and ERPF as well as significant positive correlations with ERPF (Table 3)*.* No correlations were found between RSF and the renal damage markers, PRC, or FE_Na_.

**Table 3:** Univariate association of renal hemodynamics and additional markers with RSF/TK

Variables	RSF/TK (log)^a^	*P*
GFR, mL/min/1.73 m^2^	− 0.38	**0.006**
ERPF, mL/min/1.73 m^2^	− 0.38	**0.006**
FF, %	0.18	0.215
ERVR, mmHg/L/min	0.45	**0.001**
Additional markers		
PRC (log), pg/mL	− 0.10	0.527
FE_Na_, %	0.03	0.846

Renal damage markers	RSF/TK^b^	*P*
ACR, mg/mmol	− 0.22	0.130
NGAL, ng/mmol	0.19	0.195
KIM-1, ng/mmol	0.09	0.542

Bold values indicate statistically significant correlations

*ACR* albumin/creatinine ratio, *ERPF* effective renal plasma flow, *ERVR* effective renal vascular resistance, *FE*_*Na*_ fractional excretion of sodium, *FF* filtration fraction, *GFR* glomerular filtration rate, *KIM-1* kidney injury molecule-1, *NGAL* neutrophil gelatinase-associated lipocalin, *PRC* plasma renin concentration, *RSF* renal sinus fat

^a^Data show the Pearson correlation coefficient

^b^Data show the Spearman correlation coefficient

Correlations between RSF and clinical characteristics are reported in Table 4. RSF/TK increased significantly with age (*P* = 0.004), but no other obesity-related variables including SAT, BMI, or waist circumference, although there were trends towards positive correlations with MAP (*r* = 0.28, *P* = 0.053) and VAT (*r* = 0.27, *P* = 0.056) (Table 4).

**Table 4:** Univariate associations of baseline variables with RSF/TK

Variables	RSF/TK (log)^a^	*P*
Age, years	0.39	**0.004**
Sex, (female = 1)	− 0.16	0.269
BMI (log), kg/m^2^	0.18	0.210
Waist circumference, cm	0.06	0.676
SAT (log), cm^2^	− 0.07	0.633
VAT, cm^2^	0.27	0.056
MAP, mmHg	0.28	0.053
HbA1c, mmol/mol	− 0.13	0.383
Fasting plasma glucose, mmol/L	0.10	0.507

Bold values indicate statistically significant correlations

*BMI* body mass index, *HbA1c* glycated hemoglobin, *MAP* mean arterial pressure, *RSF* renal sinus fat, *SAT* subcutaneous adipose tissue, *VAT* visceral adipose tissue

^a^Data show the Pearson correlation coefficient

Table 5 shows the regression models of RSF/TK with renal hemodynamics. GFR, ERPF, and ERVR showed associations with RSF/TK (model 1), also after adjustment for VAT (model 2), MAP, sex, and BMI (model 3). Further adjustment for age, however, rendered the influence of RSF/TK on GFR and ERPF insignificant. RSF/TK remained associated with ERVR after adjustments for all the variables (model 4).

**Table 5:** Regression models of RSF/TK with (intra-)renal hemodynamics

	Model 1	Model 2	Model 3	Model 4
		Model 1+ VAT	Model 2+ MAP, sex, BMI	Model 3+ Age
GFR (mL/min/1.73 m^2^)	− 0.37 (*P* = **0.009**)	− 0.36 (*P* = **0.008**)	− 0.33 (*P* = **0.019**)	− 0.20 (*P* = 0.205)
ERPF (mL/min/1.73 m^2^)	− 0.37 (*P* = **0.009**)	− 0.35 (*P* = **0.010**)	− 0.34 (*P* = **0.013**)	− 0.22 (*P* = 0.159)
FF (%)	0.19 (*P* = 0.196)	0.16 (*P* = 0.264)	0.22 (*P* = 0.133)	0.14 (*P* = 0.317)
ERVR (mmHg/L/min)	0.56 (*P* < **0.001**)	0.55 (*P* < **0.001**)	0.51 (*P* = **0.001**)	0.42 (*P* = **0.023**)

Multivariable linear regression analyses with RSF/TK as the dependent. Independent variables in model 1 are shown in the first column. Additional corrections are applied for VAT (model 2), MAP, sex and BMI (model 3), and age (model 4). Data show the regression coefficient *β* and (*P *values). Bold values indicate statistical significance

## Discussion

The current study is the first to assess the relation between MRI-measured RSF and renal hemodynamics as measured by gold-standard inulin (GFR) and PAH (ERPF) clearance techniques. In this cohort of T2D patients without overt CKD, we demonstrate that RSF, after correction for multiple potential confounding factors including age, VAT, MAP, sex, and BMI, is positively associated with renal vascular resistance.

Diabetic kidney disease (DKD) has become the leading cause of CKD and end-stage kidney disease (ESKD), and with the ever-increasing prevalence of obesity and T2D, this global health issue is likely to expand in the future. Despite multifactorial treatment aimed at reducing established renal risk factors (including dietary advices to reduce overweight, smoking cessation, amelioration of hyperglycemia, blood pressure- and albuminuria-lowering by blockers of the renin–angiotensin–aldosterone (RAS) system and lipid-lowering by statins) residual risk to develop ESKD remains high [23]. This highlights the need for improved insight into the pathophysiology of CKD associated with obesity and diabetes, allowing for development of novel therapeutic approaches.

In this respect, RSF has received attention in the past. Although increased fat total mass is associated with various metabolic abnormalities as well as adverse cardiorenal outcome, specific adipose tissue depots were demonstrated to convey different risks for specific diseases. For RSF, it was demonstrated that increased fat at this anatomical location is associated with hypertension as well as lower eGFR, statistically independent of fat accumulation in other compartments [7, 8]. In the present study, we add inulin clearance measurement of GFR to previous studies, which reported negative associations between RSF and eGFR (using MDRD, Cockcroft–Gault, or CKD-EPI equations), to show that increased RSF also shows a negative association with measured GFR and thus with renal function [7, 9]. These results are especially important for the T2D patients, in which eGFR has proven to be systematically underestimated [24].

Several mechanisms have been put forward by which increased fat deposition in the renal sinus may impair kidney function, beyond an increment in systemic blood pressure. First, due to compression of various renal structures, intrarenal pressure may result in less medullary perfusion and reduced tubular flow, which, in addition to inducing renal hypoxia, may stimulate increased renal sodium reabsorption [25]. In our analyses, however, no association was observed between RSF and fractional sodium excretion. Second, accumulating evidence suggests that RSF might act as a PVAT depot and like the adipocytes from increased PVAT, RSF adipocytes might exhibit altered metabolism and secrete pro-inflammatory cytokines [3]. This paracrine effect from the adipocytes could lead to renal damage through local inflammation, lipotoxicity, oxidative stress, and fibrosis [7, 14]. Although we did not measure markers of oxidative stress or urinary inflammatory cytokines, we found no association between RSF and urinary levels of kidney injury molecule (KIM)-1 or neutrophil gelatinase-associated lipocalin (NGAL). Third, RSF could modulate arterial vascular tone and renal hemodynamics by secretion of vasoconstrictive factors, in analogy with PVAT in skeletal muscle, thereby altering renal function. Renal hemodynamics have received much attention in recent decades as the two classes of drugs that provide renoprotection in T2D patients, the RAS blockers, and sodium-glucose cotransporter (SGLT)-2 inhibitors, do this by beneficially altering (intra)renal hemodynamics. In the present analysis, we could relate RSF to renal hemodynamics due to our gold-standard measured GFR and ERPF from which we calculated filtration fraction and renal vascular resistance. We observed, even after correction for VAT, MAP, sex, and BMI that higher RSF associated with increased renal vascular resistance, which likely drives the lower GFR at the hemodynamic level. Estimated afferent arteriolar pressure shows a similar association with RSF. The increment in renal vascular resistance is relevant as this parameter, often estimated with Doppler ultrasonography-derived renal resistance index, has been shown to be associated with albuminuria and predicted progression of CKD in hypertensive patients [26]. The mechanism by which RSF could modulate renal arteriolar resistance is unsure; however, a decrease in adiponectin production, probably due to the increased pro-inflammatory effects of macrophages or due to adipose tissue insulin resistance, leads to reduced ability to relax smooth muscle tonus [14]. In addition, the production of the vasorelaxing factors such as nitric oxide (NO) by endothelial cells may be decreased by the altered secretion of PVAT adipocytes [14], which could increase renal afferent resistance. Finally, it has been suggested that increased RSF leads to hypertension by activating RAS, possibly by mechanical compression of the low-pressure structure of the renal hilum [9]. We, however, observed no association between RSF and efferent vascular resistance, where angiotensin II—the final product of RAS and a potent vasoconstrictor—is known to mediate its effects. In line, we could not link RSF with plasma renin levels, although the extensive use of RAS blockers in our group may have confounded these results. Due to the relatively small number of participants in our study population, subgroup analyses between the RAS-blocker group and the group not on RAS blockers did not provide us with reliable outcomes.

A significant strength of our study is the gold-standard methods for calculations of GFR and ERPF. On the other hand, this analysis has some limitations that need to be mentioned. First, the cross-sectional design of the study does not allow to address causality regarding the effects of RSF on renal hemodynamics. Second, our research was limited to the assessment of the quantity of the RSF, rather than the quality (i.e., possible biological/paracrine effects as observed for PVAT adipocytes). Therefore, from our results, we cannot draw conclusions about this possible paracrine regulation of vascular tone of RSF. Moreover, it would be interesting to repeat this research in a larger study population, to measure both kidneys and preferably use the Dixon technique to generate fat-only images from multi-echo MRI images for even more accurate fat quantification.

Further studies that modulate the amount of RSF, notably weight loss are warranted [27], however, it will be difficult to tease out the specific effects of a reduction in RSF volume on renal function, when VAT and total body fat are concomitantly lowered. Furthermore, it will be interesting to measure proteins and cytokines that are secreted by RSF in different disease conditions such as obesity or T2D to better assess RSF function.

In conclusion, our results in T2D patients without overt CKD indicate that excess of RSF is associated with altered renal hemodynamics most notably with increased renal vascular resistance. As increased ERVR is associated with CKD and CKD progression, modulating RSF may provide a novel therapeutic approach to reduce the DKD burden.

## Electronic supplementary material

Below is the link to the electronic supplementary material.

Supplementary file1 (DOCX 157 kb)
