# Supplementary material for: Renal sinus fat and renal hemodynamics: a cross-sectional analysis
Source: MAGMA. 2019 Aug 31;33(1):73–80. doi: 10.1007/s10334-019-00773-z (PMC7021744; doi:10.1007/s10334-019-00773-z)
Supplement: Supplementary file 1 — Supplementary file1 (DOCX 157 kb) [file 10334_2019_773_MOESM1_ESM.docx]

# Supplementary materials

## Validation single slice approximation of renal sinus fat

### Methods

As explained in the methods section, 15 patients of whom the entire left kidney was imaged were selected for this validation. For those subjects, a ROI encompassing the fatty tissue within the renal sinus was manually segmented on all slices. This measurement is denoted as renal sinus fat (RSF). For the single slice estimation, the slice where the renal artery entered the kidney was used, we will denote this the single slice RSF (ssRSF). A scatterplot and Pearson’s correlation coefficient were used to assure a linear correlation between both.

However, to assess whether the eRSF is a good representation of the RSF, we have to look into agreement rather than correlation. Although a Bland-Altman approach would be appropriate, it is not straightforward due to the large difference in magnitude between the ssRSF (average 35.5 cm^3^) and RSF (average 1.44 cm^3^) Therefore, we fitted a linear model of the form:

$$RSF\approx\alpha*ssRSF+\beta$$

This enables us to perform a Bland-Altman analysis using RSF and eRSF, where RSF is the reference standard.

### Results

A scatter plot of RSF versus ssRSF is shown in figure S1. A Pearson’s correlation coefficient of 0.78 was obtained (P<0.001). For the linear model to express eRSF in terms of ssRSF we found coefficients α 0.029 and β 0.397, so:

$$eRSF\approx20.5*ssRSF+6.0$$

Here, eRSF is the estimated RSF based on the ssRSF. R^2^ was 0.60 and P<0.001.

The Bland Altman analysis for RSF and eRSF yielded the scatter plot and Bland Altman plot in figure S2 and S3. For the Bland Altman plot, on the x-axis the RSF instead of the mean of RSF and eRSF was used, because RSF was considered the reference standard.

There was no bias between RSF and eRSF, but we can appreciate that for smaller values of RSF, eRSF tends to overestimate RSF. The opposite is true for large values of RSF: the eRSF tends to underestimate RSF. The same can be appreciated in the scatter plot (figure S3).

### Conclusion

eRSF and ssRSF can be considered equivalent in the analyses presented in the main manuscript, since they are linearly related. For linear regression as performed in the main manuscript, it does not matter whether ssRSF or eRSF is used for the significance of the correlations. Therefore, limitations of the eRSF are also limitations of the ssRSF.

Therefore, we can conclude from this analysis that ssRSF can be used to estimate RSF, but that the RSF is overestimated for small RSF and underestimated for large RSF values.

| 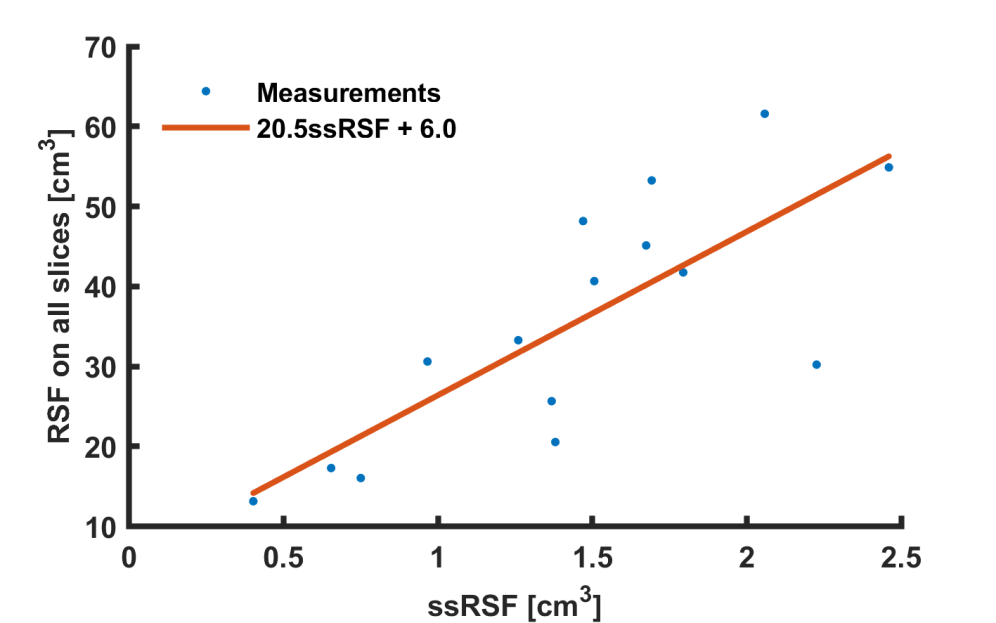 |
| --- |
| **Figure 1:** Scatterplot of RSF versus ssRSF, with the best fit. |
| 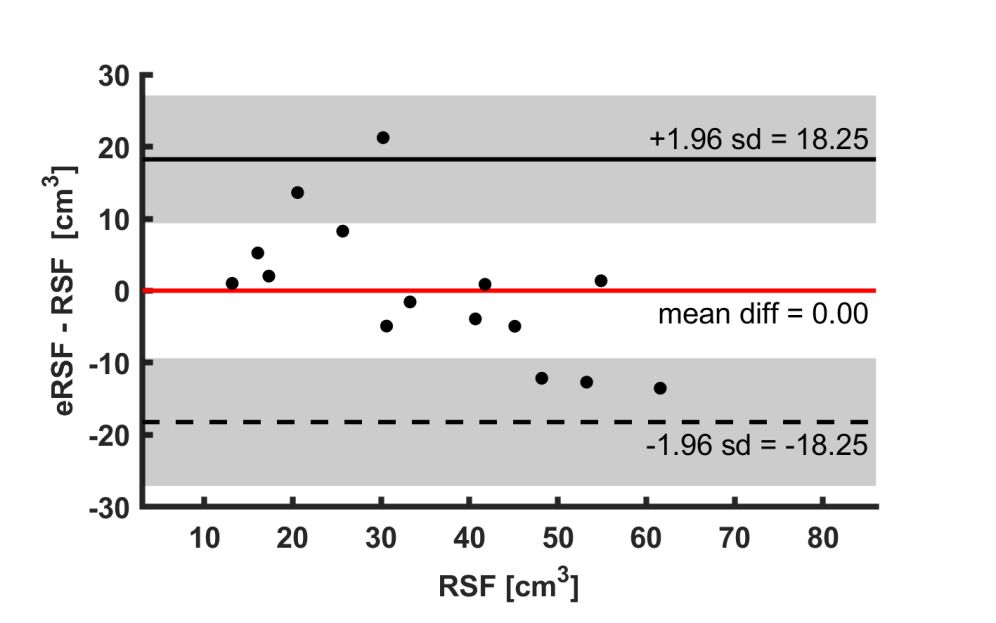 |
| **Figure 2:** Bland Altman plot showing the difference between eRSF and RSF versus the reference standard (RSF). The slight overestimation of RSF for low values of RSF and the underestimation of RSF for higher values of RSF can be clearly appreciated. Shaded areas denote the confidence intervals of the limits of agreement. |
| 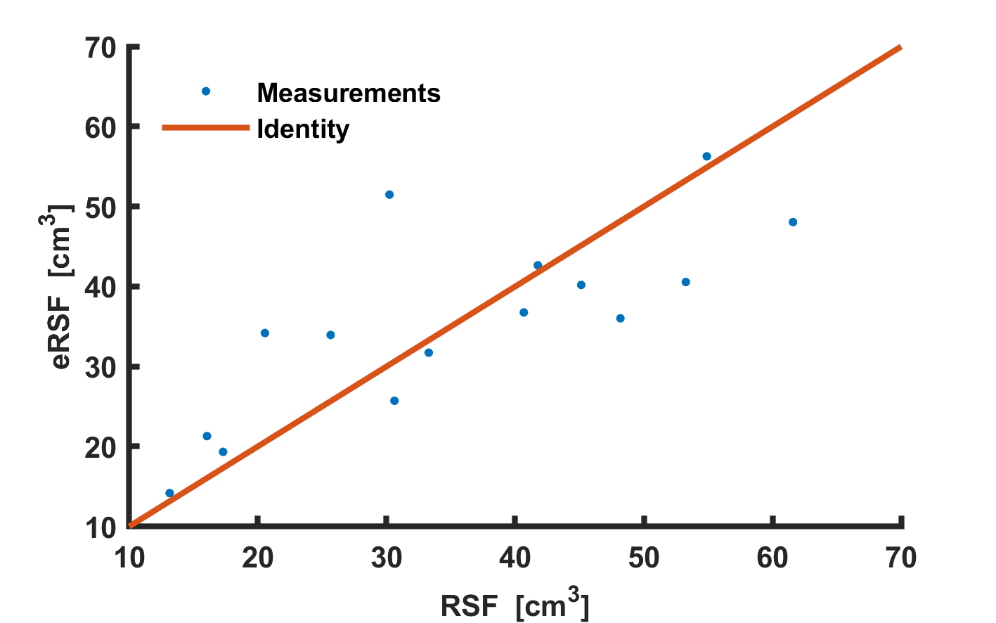 |
| **Figure 3:** Scatterplot of eRSF vs RSF, with the line of identity. |
